# Airflow and Particle Transport Prediction through Stenosis Airways

**DOI:** 10.3390/ijerph17031119

**Published:** 2020-02-10

**Authors:** Parth Singh, Vishnu Raghav, Vignesh Padhmashali, Gunther Paul, Mohammad S. Islam, Suvash C. Saha

**Affiliations:** 1 School of Mechanical and Mechatronic Engineering, University of Technology Sydney (UTS), 15 Broadway, Ultimo, NSW 2007, Australia; 13305382@student.uts.edu.au (P.S.); 13132232@student.uts.edu.au (V.R.); 13121443@student.uts.edu.au (V.P.); mohammadsaidul.islam@uts.edu.au (M.S.I.); 2 James Cook University, Australian Institute of Tropical Health and Medicine, Townsville, QLD 4810, Australia

**Keywords:** airflow, airway stenosis, lung, COPD, airway particle transport, respiration

## Abstract

Airflow and particle transport in the human lung system is influenced by biological and other factors such as breathing pattern, particle properties, and deposition mechanisms. Most of the studies to date have analyzed airflow characterization and aerosol transport in idealized and realistic models. Precise airflow characterization for airway stenosis in a digital reference model is lacking in the literature. This study presents a numerical simulation of airflow and particle transport through a stenosis section of the airway. A realistic CT-scan-based mouth–throat and upper airway model was used for the numerical calculations. Three different models of a healthy lung and of airway stenosis of the left and right lung were used for the calculations. The ANSYS FLUENT solver, based on the finite volume discretization technique, was used as a numerical tool. Proper grid refinement and validation were performed. The numerical results show a complex-velocity flow field for airway stenosis, where airflow velocity magnitude at the stenosis section was found to be higher than that in healthy airways. Pressure drops at the mouth–throat and in the upper airways show a nonlinear trend. Comprehensive pressure analysis of stenosis airways would increase our knowledge of the safe mechanical ventilation of the lung. The turbulence intensities at the stenosis sections of the right and left lung were found to be different. Deposition efficiency (DE) increased with flow rate and particle size. The findings of the present study increase our understanding of airflow patterns in airway stenosis under various disease conditions. More comprehensive stenosis analysis is required to further improve knowledge of the field.

## 1. Introduction

Ambient air pollution is a global problem which has affected all countries across the world. Developing countries particularly suffer from environmental air pollution, as they often derive inexpensive energy from burning natural fossil resources. However, they do not have access to technologies to mitigate potential air pollution. Air pollution is an important environmental risk factor with global health implications. The respiratory system becomes a target of harmful air pollutants, including ozone, nitrogen dioxide, volatile organic compounds, and particulate matter, affecting the lungs specifically [1]. Lung functionality normally decays with age; however, air pollution is contributing to breathing problems and acute or chronic lung diseases [2]. The overall impact of exposure to pollutants on lung health and the treatment of respiratory diseases have become of increased interest to researchers. Our knowledge and understanding of the impact of ambient air pollution on the human lung has been improved by computerized modelling. 

A wide range of in silico [3,4,5,6,7,8,9,10] and experimental [11,12] studies have been performed on airflow and particle transport characterization in idealized and realistic models of the lung. Almost all the available literature, however, considers healthy airways for airflow and particle transport modelling. Yet, volume reduction of the respiratory airways occurs due to age and disease conditions, which creates airflow limitations [13]. Respiratory diseases like asthma also create airway inflammation and obstruct the airway [14,15,16]. A number of analytical and experimental studies have analyzed functional abnormality of the nasal and tracheal airways of the lung. A clinical study of fluid flow through nasal obstruction concluded that nasal pyriform could potentially help vestibular reduction in the nasal cavity [17]. An in situ study of the laryngeal airway investigated airway resistance by tracing flow pressure and reported no connection between local resistance and dyspnea score [18]. Recently, a study discussed pressure variation and corresponding flow behavior in a stenosis section of the trachea [19]. Their calculation showed that the flow velocity for 1 mm tracheal diameter was about 100 m/s, which seems rather high in real life. A numerical study reported a pressure drop and energy loss at the tracheal stenosis section during inhalation and exhalation [20]. The study did not consider the oral airway and reported the maximum velocity at the stenosis section. The pressure drop during inhalation was found to be higher than that for exhalation. While all the above studies improved our knowledge of airflow in the oral airway and trachea of the lung, they did not consider particle transport through a stenosis section. Inthavong et al. performed a detailed analysis of airway obstruction for an asthma patient [21]. This study used a CT-based realistic model of the tracheal obstruction and calculated the particle deposition efficiency (DE). However, airway volume reduction can happen at the tracheobronchial airways of the right and left lung under various diseases conditions [22], and a precise understanding of airflow and particle transport in stenosis sections of both the right and left lung would enrich knowledge in this field. A number of other studies [23,24,25,26,27] have performed both airflow and particle transport analysis for large-scale models; however, they did not consider stenosis in the tracheobronchial airways. 

This study focuses on abnormality of the pulmonary airways in the right and left lung. We analyze the details of airflow dynamics and particle transport through stenosis sections of the right and left lung. 

## 2. Numerical Method

Mouth–throat and upper airway models were reconstructed from digital CT images. Three different anatomical models were generated for the comparison. Stenosis sections were reconstructed in the modelling software SolidWorks 2019 (DASSAULT SYSTEMES SolidWorks Corp., Waltham, MA, USA). Fluid flow and particle transport equations were solved through conservation of mass and momentum assumptions:

(1) $$\frac{\partial \rho }{\partial t}+\nabla \cdot \left(\rho \vec{v}\right)=S_{m}$$

where *S_m_* is the mass source term, and

(2) $$\frac{\partial }{\partial t}\left(\rho \vec{v}\right)+\nabla \cdot \left(\rho \vec{v}\vec{v}\right)=-\nabla p+\nabla \cdot \left(\mu \left[\left(\nabla \vec{v}+\nabla {\vec{v}}^{Τ}\right)-\frac{2}{3}\nabla \cdot \vec{v}I\right]\right)+\rho \vec{g}+\vec{F}$$

where *p* is fluid static pressure, $\rho \vec{g}$ is body force due to gravity, and $\vec{F}$ is body force due to external (particle–fluid interaction) force.

The k–ϵ turbulence model was considered as a viscous model. The equations for the calculation of turbulent kinetic energy and dissipation rate in an inertial frame are as follows.

For turbulent kinetic energy *k*,

(3) $$\frac{\partial \left(\rho k\right)}{\partial t}+\frac{\partial \left(\rho ku_{i}\right)}{\partial x_{i}}\;=\;\frac{\partial }{\partial x_{j}}\left[\frac{\mu }{\sigma_{k}}\;\frac{\partial k}{\partial x_{j}}\right]+2\mu_{t}E_{ij}E_{ij}-\rho$$

and for dissipation,

(4) $$\frac{\partial \left(\rho \varepsilon \right)}{\partial t}+\frac{\partial \left(\rho \varepsilon u_{i}\right)}{\partial x_{i}}\;=\;\frac{\partial }{\partial x_{j}}\left[\frac{\mu_{t}}{\sigma_{\varepsilon }}\;\frac{\partial \varepsilon }{\partial x_{j}}\right]+\varepsilon_{1\varepsilon }\frac{\varepsilon }{k}2\mu_{t}E_{ij}E_{ij}-\varepsilon_{2\varepsilon }\rho \frac{\varepsilon^{2}}{k}$$

where $u_{i}$ represents the velocity component in the corresponding direction, $E_{ij}$ represents the component of the rate of deformation, and $\mu_{t}$ represents eddy viscosity.

The equations also consist of some adjustable constants $\sigma_{k}$, $\sigma_{\varepsilon }$, $C_{1\varepsilon }$, and $C_{2\varepsilon }$ The values of these constants were derived through numerous iterations of data fitting for a wide range of turbulent flows. These constants are

$$C_{\mu }=0.09,\;\sigma_{k}=1.00,\;\;\sigma_{\varepsilon }=1.30,\;C_{1\varepsilon }=1.44,\;\;C_{2\varepsilon }=1.92.$$

Air is considered the primary fluid at 1.225 kg/m^3^ density and 1.7893 × 10^−5^ kg/ms viscosity, whereas aerosol of 1100 kg/m^3^ density is considered as the secondary phase in the discrete phase model. A surface injection method was employed, and particles were introduced through the face normal direction of the mouth–throat inlet surface. 

The chosen k–epsilon model incorporates modifications of a scalable wall function near where the walls interact with continuous flow, improving the predictions of the spreading rate for planar, spherical, or round surfaces. Two transport variables are prescribed for turbulent length scales for moderate- to high-complexity flow in the system, one being turbulent kinetic energy (*k*) and the other the dissipation rate of turbulent kinetic energy (*ε*). This model is most suited for free-shear layer flows along relatively small pressure gradients. It accommodates the initial and boundary conditions supplied for the calculations, making it the simplest and most convenient model. Pressure–velocity coupling [28] and second-order upwind discretization techniques [29] were used for the numerical calculations. The following particle transport equation was solved:

(5) $${\vec{F}}_{D,i}=\frac{1}{2}C_{D}\frac{\pi d_{p,i}^{2}}{4}\rho \left({\vec{v}}_{p,i}-\vec{v}\right)\left|{\vec{v}}_{p,i}-\vec{v}\right|$$

where $C_{D}$ and *d_p_* are the drag coefficient and particle diameter, respectively, and ${\vec{v}}_{p}$ is the particle velocity. The single-particle motion *i* was modelled using Newton’s second law:

(6) $$m_{p,i}\frac{\partial {\vec{v}}_{p,i}}{\partial t}={\vec{F}}_{D,i}+m_{p,i}\vec{g}.$$

The numerical investigation was performed for monodisperse particles, and particles were injected from the mouth–throat surface. Particles of diameter 2.5, 5, and 10 µm were injected at three different flow rates of 7.5, 15, and 30 lpm (litre per minute). Boundary conditions were taken as the inlet velocity and outlet outflow. Wall conditions of stationary walls and no slip were used. A DPM (Discrete Phase Model) wall boundary condition was used as a trap, and a heat flux thermal condition was used at the wall. Airway dynamic motion was ignored for the present model as the available literature shows a negligible impact on particle transport. 

## 3. Geometrical Development

**Figure 1:**
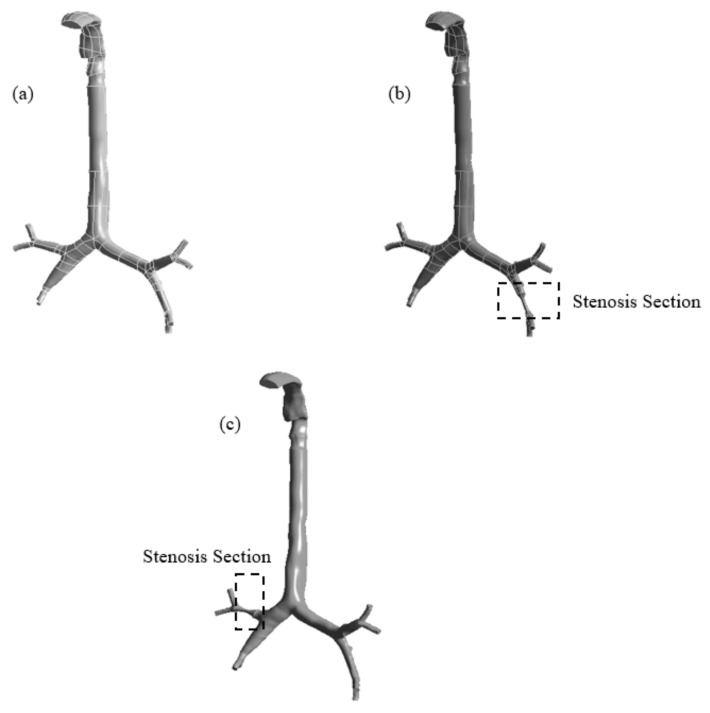
Reconstructed models of the mouth–throat and tracheobronchial airways: (**a**) healthy, (**b**) stenosis airway in the right lung, and (**c**) stenosis airway in the left lung.

This study employed digital CT images for the airway anatomical model. The lung geometry comprises two CT scan models of the mouth–throat and upper airways of a 50-year-old man. The mouth–throat section was assembled with the tracheobronchial airways. Figure 1 shows the reconstructed anatomical model for three different health conditions. Two stenosis anatomical models were developed using SolidWorks. Three different models were created for a similar number of generations. One model represents the normal healthy lung condition with no abnormal conditions, while the other two geometries represent pulmonary stenosis, one on the left lobe and the other on the right lobe. Pulmonary stenosis involves squeezing of the artery, reducing the lobe to 25% of its original diameter. Figure 1a shows the healthy airway model with no airway reduction. Figure 1b,c shows the stenosis sections at the selected positions of the left and right lung, respectively. The stenosis section was developed with a smooth wall surface. 

## 4. Grid Generation and Model Validation

**Figure 2:**
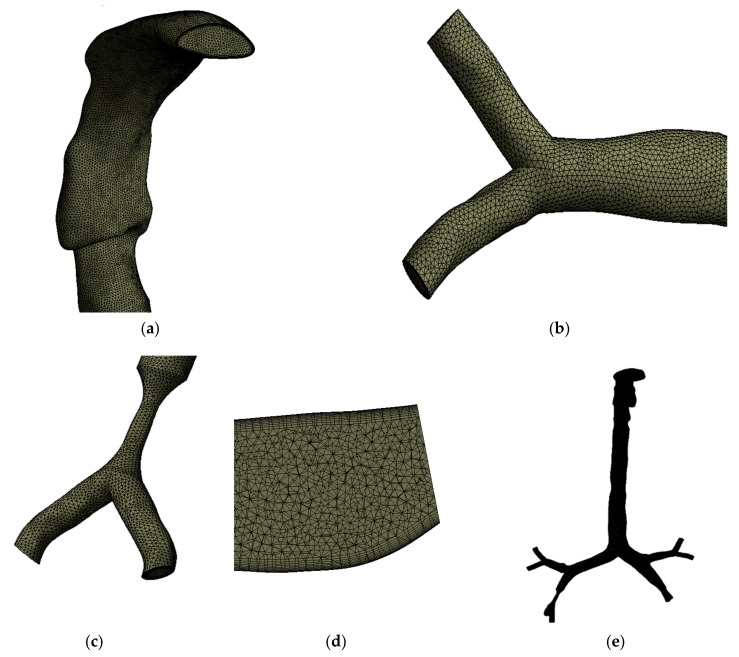
Unstructured mesh for the model, (**a**) the mouth–throat, (**b**) the mesh at a bifurcating branch, (**c**) the mesh at the stenosis area, (**d**) a cross section of the inflation layer mesh, and (**e**) the complete computational model.

The anatomical models of the healthy and stenosis airways consist of a highly complex asymmetric branching pattern. Unstructured mesh elements were generated for all three models. Figure 2 shows the mesh at different sections of the airway. Figure 2a,b shows the generated tetrahedral elements at the mouth–throat and third bifurcation of the airway, respectively. Figure 2c shows the generated mesh at the stenosis section of the left stenosis model. A lesser number of elements are presented for better visualization of the mesh type at the stenosis section. The design and calculation methods were adopted from Islam et al. [23], which describes a 17-generation design of a similar model, though more refined. All scans and geometries created in SolidWorks were combined and converted to solid geometry. The ANSYS meshing tool was used for mesh generation. Adaptable sizing and inflation for all the asymmetric regions were used to avoid contradicting voids and spaces. Figure 2d shows a cross-sectional view of the inflation layer mesh. Inflation generation was required for the geometry as bends or irregularity in the design might affect particle movement inside the body.

**Figure 3:**
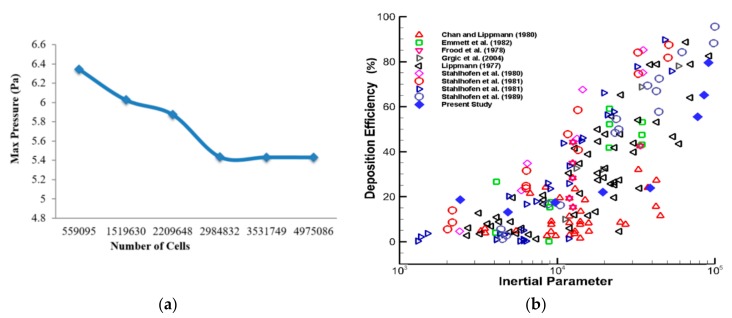
Grid refinement and model validation: (**a**) mesh-independent (maximum pressure calculated at the selected plane in the mouth–throat area) under the 30 lpm inlet condition, and (**b**) deposition efficiency as a function of the inertial parameter ρd_p_^2^Q (gµm^2^s^−1^) at the mouth–throat region; our simulation result and results from literature [30,31,32,33,34,35,36,37].

Following the patch conforming method, a sizing tool was used to generate the different mesh cells for all the velocities processed. All three geometries were processed in mesh generation under a fluent solver condition. The geometry consists of various regions with irregularities and spontaneous surface changes, which makes grid selection very difficult. In this case, inflation generation was carried out on one of the bifurcations with a certain bend, to avoid particles being trapped along with movement away from the wall. Figure 2e shows the whole model using tetrahedral elements; elements are not visible due to the large number of computational elements. After carrying out the grid refinement test, subject to several designs, about 3.53 million elements were found to be stable. A number of different mesh elements were tested against the maximum pressure at a selected plane of the mouth–throat area. Figure 3a shows the grid-independent test results. The minimum orthogonal quality was checked for all cases, and the average quality for all models was found to be 0.34.

Simulated study results from the numerical model were validated using benchmark experimental measurements. The DE at the mouth–throat area was compared with that in the available literature [30,31,32,33,34,35,36,37]. The DE at the oral airways is shown as a function of the inertial parameter ρdp2Q (g·µm^2^·s^−1^) and presented in Figure 3b. The DE of the numerical simulation was found to be in the range of that in the published literature, confirming that the model is precise enough for further analysis. 

## 5. Results and Discussion 

**Figure 4:**
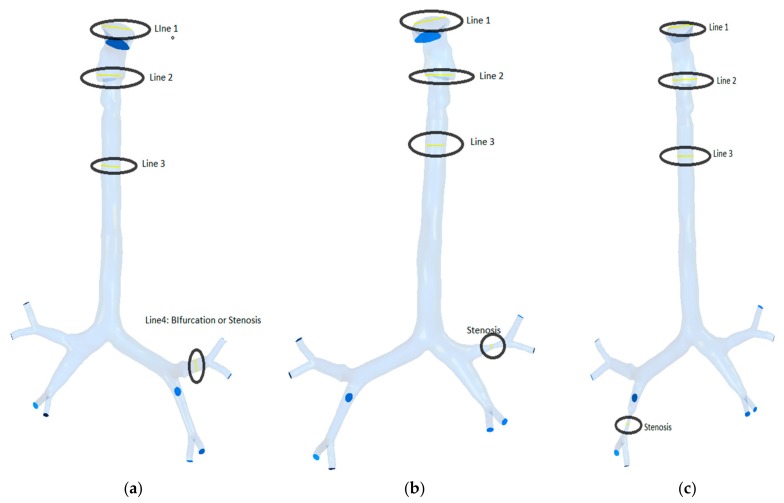
Selected lines for velocity profile: (**a**) healthy model, (**b**) right stenosis, and (**c**) left stenosis.

The presented model was used to study airflow and particle transport at three different flow rates. The study was performed for monodisperse particle transport in the bifurcating airway (Figure 4). Calculations were carried out at three flow rates of 7.5, 15, and 30 lpm for different particle sizes.

### 5.1. Airflow Analysis

**Figure 5:**
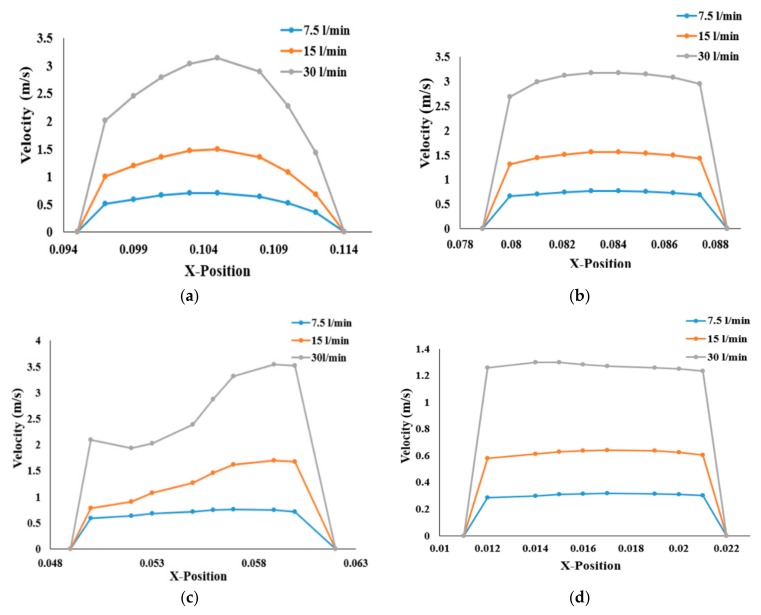
Velocity profiles for the healthy lung and three different flow rates, at (**a**) Line 1, (**b**) Line 2, (**c**) Line 3, and (**d**) at the third generation.

The airflow velocity profiles for various breathing patterns are plotted at four different cross sections of the three geometries in Figure 4. The velocity profiles at the mouth–throat sections (Lines 1 and 2), trachea (Line 3), and stenosis section (Line 4) of the bifurcating airway portions are plotted for better understanding of the flow field. Figure 5 shows the airway velocity profiles at the various positions of healthy airways. At the mouth–throat area (Figure 5a), velocity profiles are parabolic, and the flow field is fully developed for all inhalation cases. At the lower sections of the mouth–throat and tracheal area, the velocity becomes locally transitional. Figure 5b,c shows the transitional behavior of the velocity profile at Line 2 and Line 3 of the healthy lung. The velocity profiles indicate a logarithmic proportionality of higher flow rates through airflow velocity, which is quasi-linear for smaller flow rates and increases significantly for higher flow rates. It is evident that flow could be locally transitional at the tracheal wall region [38], and the pressure-driven force and strong change in airway curvature influence the flow pattern in the central airways. As can be seen in Figure 5, airflow velocity is higher in the throat region compared to the third generation. Airflow velocity at the third bifurcation (Line 4) of the healthy lung is reduced by a factor of 1:2.5 compared to that at the upper airways, as shown in Figure 5d. 

**Figure 6:**
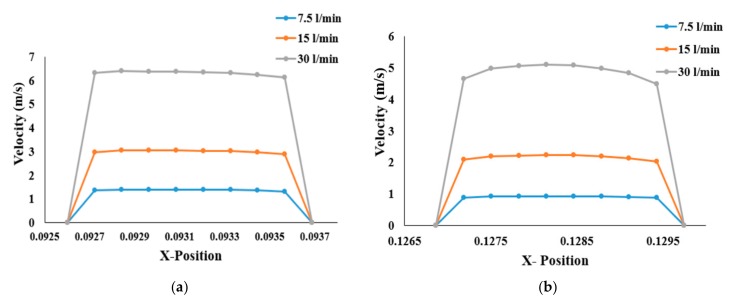
Velocity profiles at the stenosis section of the airway: (**a**) right stenosis and (**b**) left stenosis models.

Compared to those of the healthy lung, velocity profiles for stenosis conditions are different in the left and right stenosis portions of the lungs. Airflow velocity at the right stenosis area rises significantly (Figure 6a) for all flow rates compared to that in the healthy airway model. Similar behavior can be seen at the left stenosis for all flow conditions. Irrespective of the various flow rates, airflow velocity at the right stenosis is higher than at the left stenosis. The sudden rise in airflow velocity is due to the constriction of the pulmonary artery to 25% of its normal diameter. The law of mass conservation determines that air must then flow much faster than its normal flowrate due to the smaller volume. The higher airflow velocity in the right lung compared to the left lung is due to airflow distribution in the lungs. The left lung is smaller and narrower than the right lung, causing more airflow resistance; thus, more air flows to the right lung than the left lung (see [23,39]). The higher airflow distribution to the right lung increases the airflow velocity at the stenosis section of the right lung over that of the left lung. 

**Figure 7:**
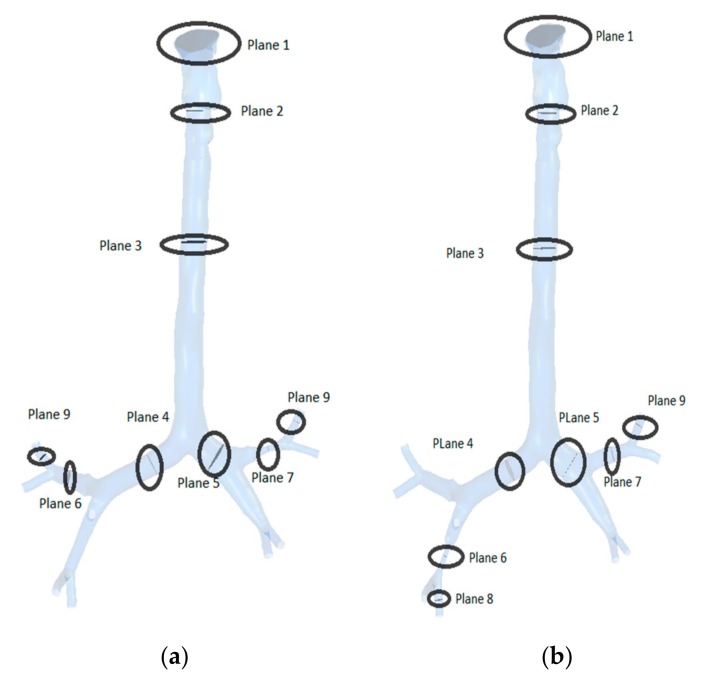
Selected planes for different lung stenosis models: (**a**) stenosis of the right lung and (**b**) stenosis of the left lung.

**Figure 8:**
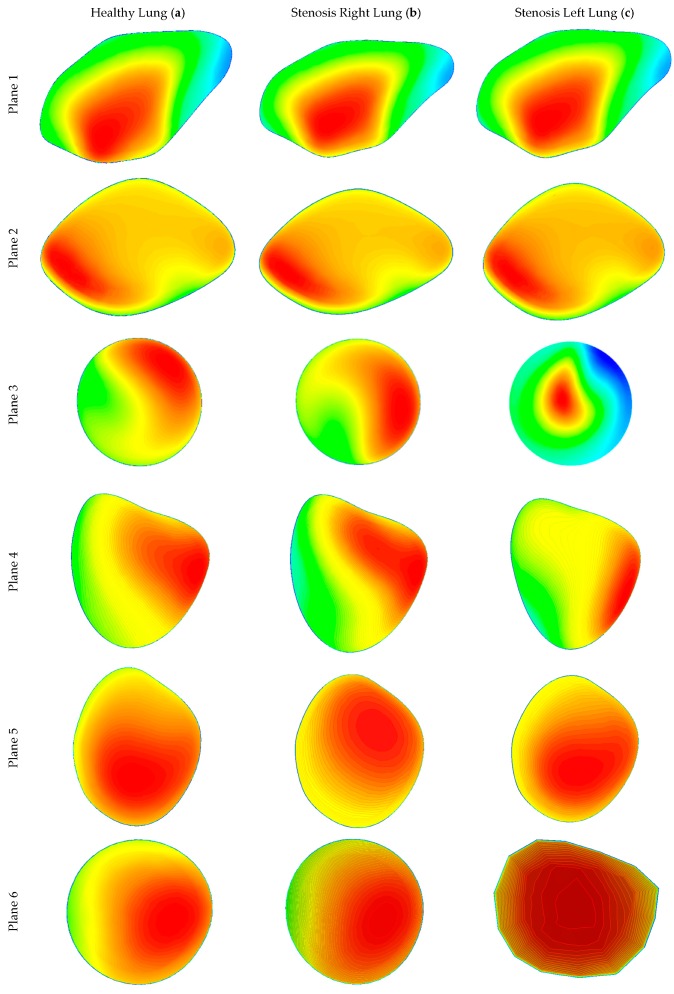

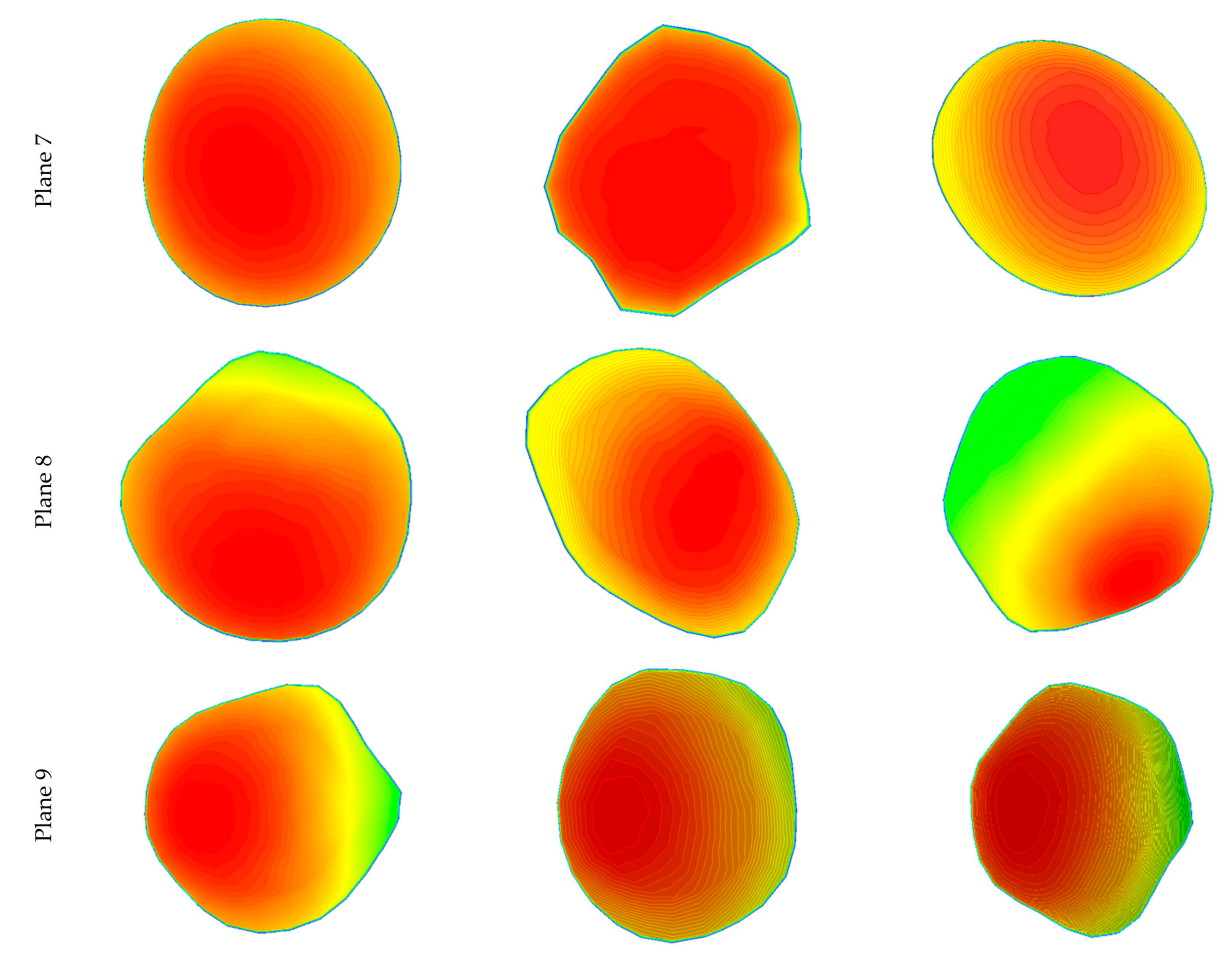
Velocity contours at different positions of the healthy and stenosis airways at 30 lpm: (**a**) healthy model (left panel), (**b**) stenosis on the right, and (**c**) stenosis on the left.

Various planes were created at different locations in the three lung models to calculate airflow velocity contours during inhalation. Figure 7 shows the plane locations starting from the mouth, via the throat, and all the diversions and the stenosis sections in the respective geometries. Airflow velocity contours at these nine different positions were calculated for healthy and stenosis airways, as presented in Figure 8. The airflow velocity field at the mouth–throat section of all three models shows a similar flow pattern along the anatomical shape of the mouth–throat, which does not differ for the healthy and stenosis airways. Airflow velocity contours at the upper sections of the airway are similar; however, the anatomical differences and shapes of the right and left stenosis then influence the flow patterns. Airflow velocity contours at the stenosis sections and beyond were found to be different for all models. Pressure-driven force, a strong change in airway curvature, the asymmetric airway shape, and turbulence fluctuation at the stenosis section affect the airflow velocity contours. Airflow velocity contours differ significantly between the right and left lung from Plane 3 onwards. 

### 5.2. Pressure Drop and Wall Shear

**Figure 9:**
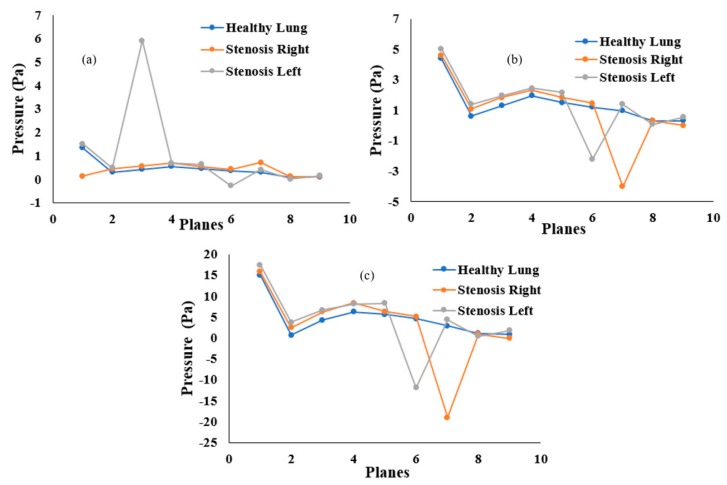
Pressure drop at different positions of the mouth–throat and upper airway for the healthy and stenosis lungs at (**a**) 7.5 lpm, (**b**) 15 lpm, and (**c**) 30 lpm.

Airway pressure plays an important role in breathing. The airway pressure has to be lower than atmospheric pressure as air flows from the higher-pressure zone to the lower-pressure zone. This numerical study investigated the airway pressure throughout the mouth–throat and upper airways. The pressure drop at different positions was calculated for the healthy and stenosis airways. Figure 9 presents the overall pressure drop at selected planes for different inhalation conditions. At a low flow rate of 7.5 lpm, the pressure drop throughout the healthy and stenosis airways shows a negligible difference between models, except for the mouth–throat area. A small pressure drop was observed at the left stenosis. At 15 lpm, the highest pressure was found at Plane 1 in all three models. In the healthy lung, pressure drops steadily from Plane 4, while pressure drops drastically at the left and right stenosis. A similar pattern was observed for the 30 lpm flow rate. Pressure drop at the right stenosis was found to be higher than that at the left stenosis. Lower pressure at the stenosis section increases airway resistance, which eventually makes inhalation more difficult. 

**Figure 10:**
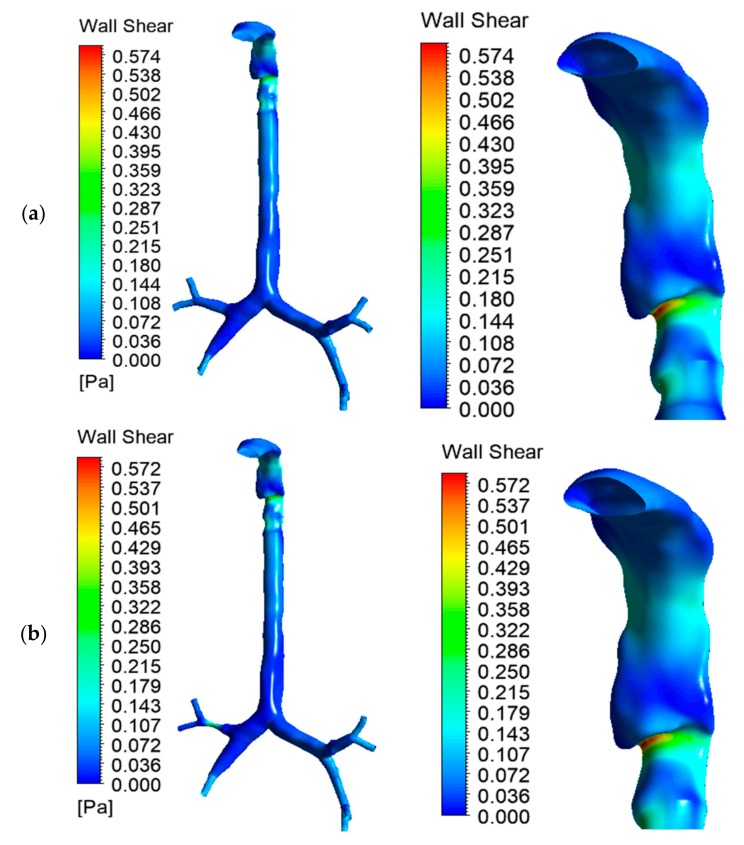

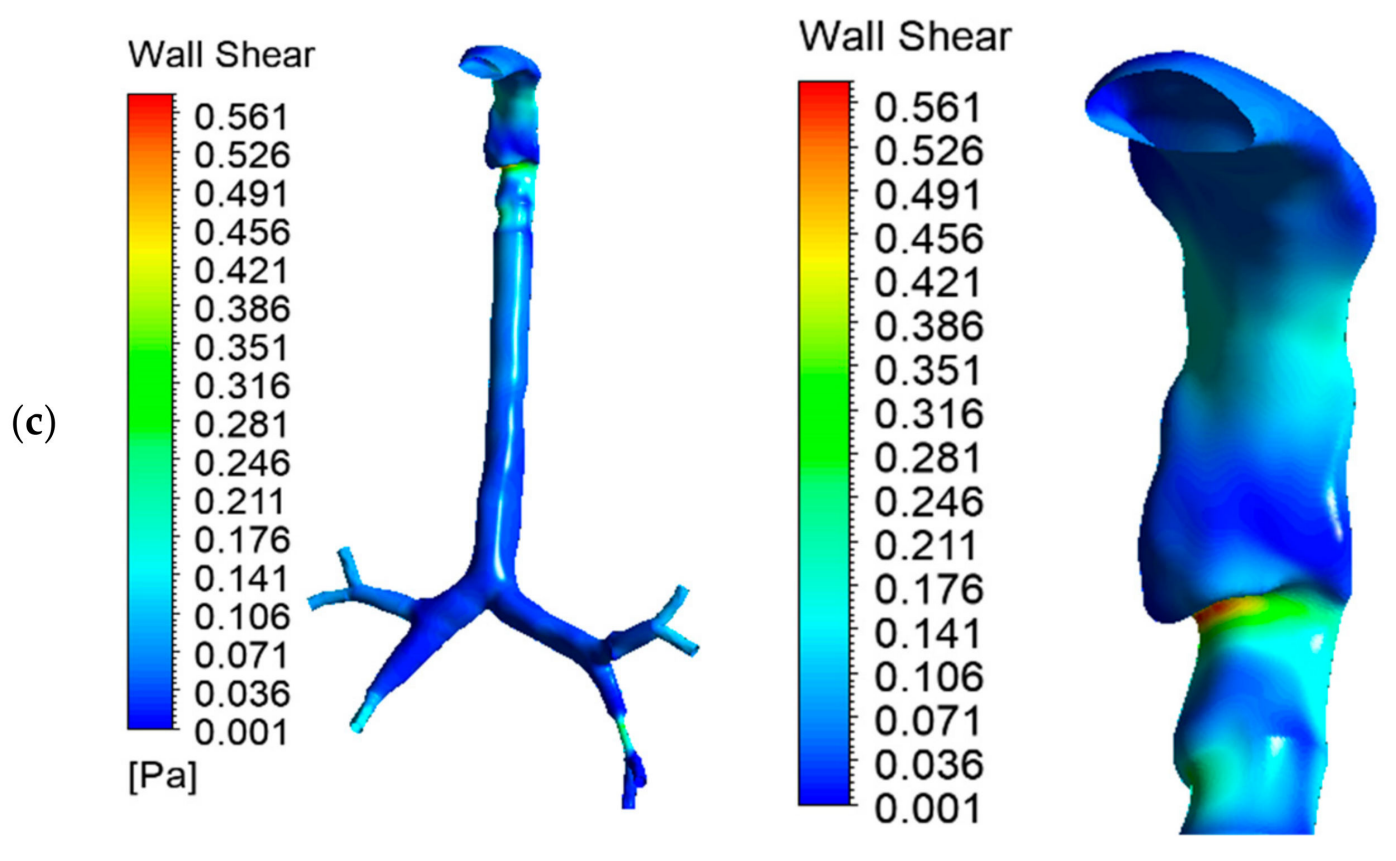
Wall shear for the three lung models at 30 lpm: (**a**) healthy lung, (**b**) left stenosis lung, and (**c**) right stenosis lung. The left panel shows wall shear for the whole model; the right panel shows wall shear at the mouth–throat.

Wall shear at the mouth–throat and tracheobronchial airway was calculated for the healthy and stenosis models. Figure 10a–c presents the wall shear contours at 30 lpm for healthy lung, right stenosis, and left stenosis, respectively. All three models depict similar wall shear at the mouth–throat region. Wall shear is a velocity-dependent force acting on the solid airway wall due to the motion of bypassing fluid. Wall shear stress is highest behind the laryngeal mouth–throat passage and at stenosis, as well as later passages into higher generations of bronchi. The models were simplified insofar as mucous fluid present in the airway was not considered; this may affect air movement inside the lungs. 

### 5.3. Turbulence Intensity

**Figure 11:**
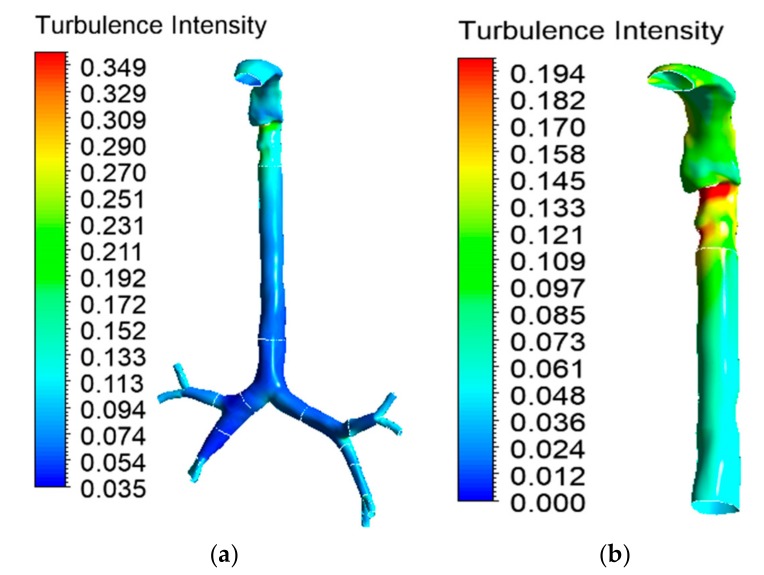

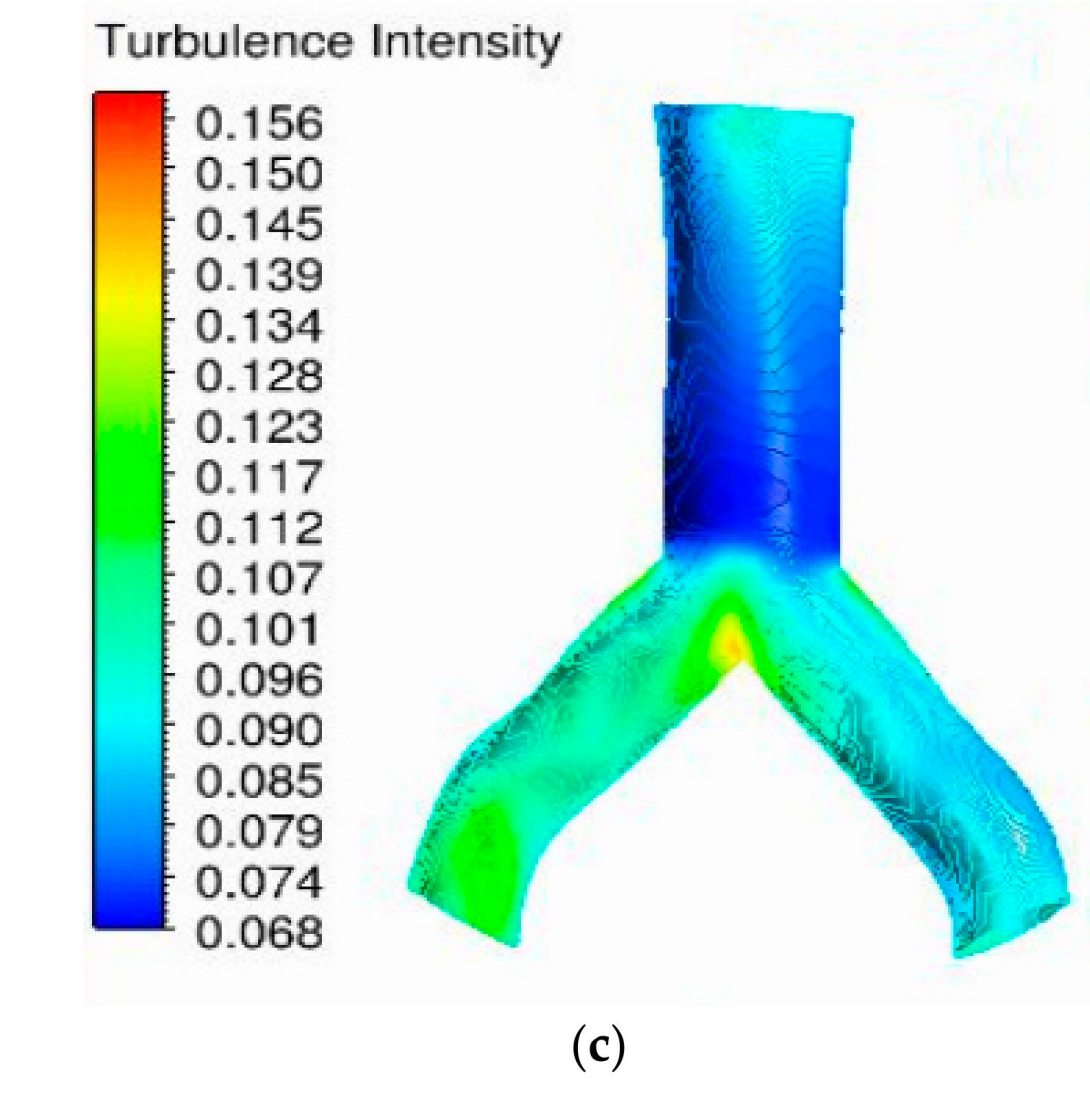
Turbulence intensity at 30 lpm for the healthy lung model: (**a**) whole model, (**b**) mouth–throat and trachea (intensity range rescaled), and (**c**) third bifurcation of the healthy lung.

**Figure 12:**
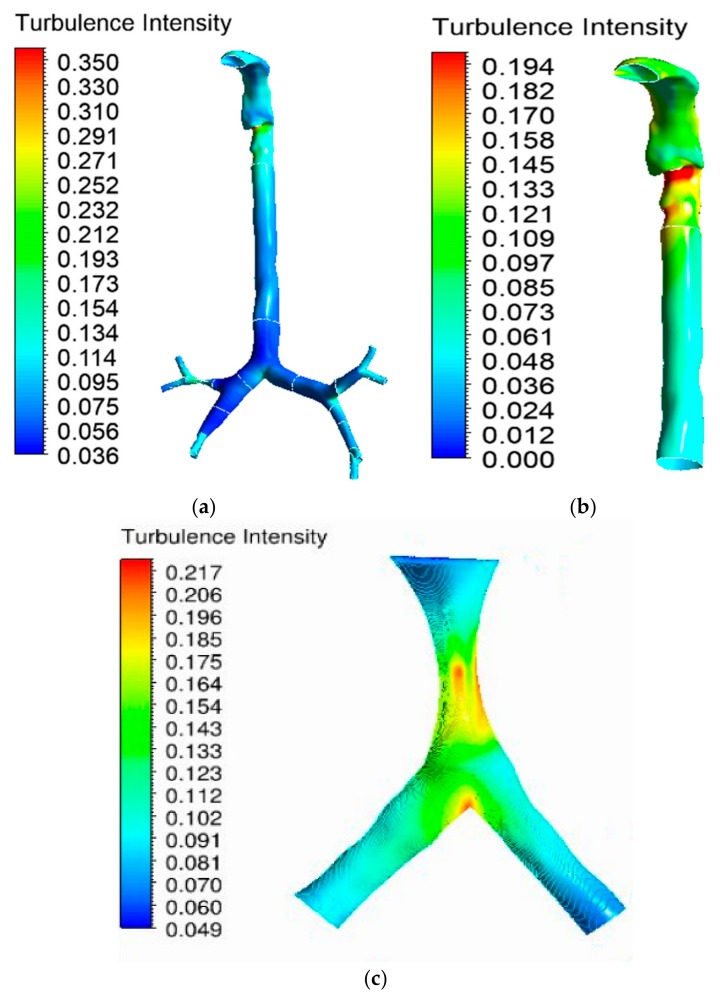
Turbulence intensity at 30 lpm for the left stenosis lung model: (**a**) whole model, (**b**) mouth–throat and trachea (intensity range rescaled), and (**c**) left lung stenosis area.

**Figure 13:**
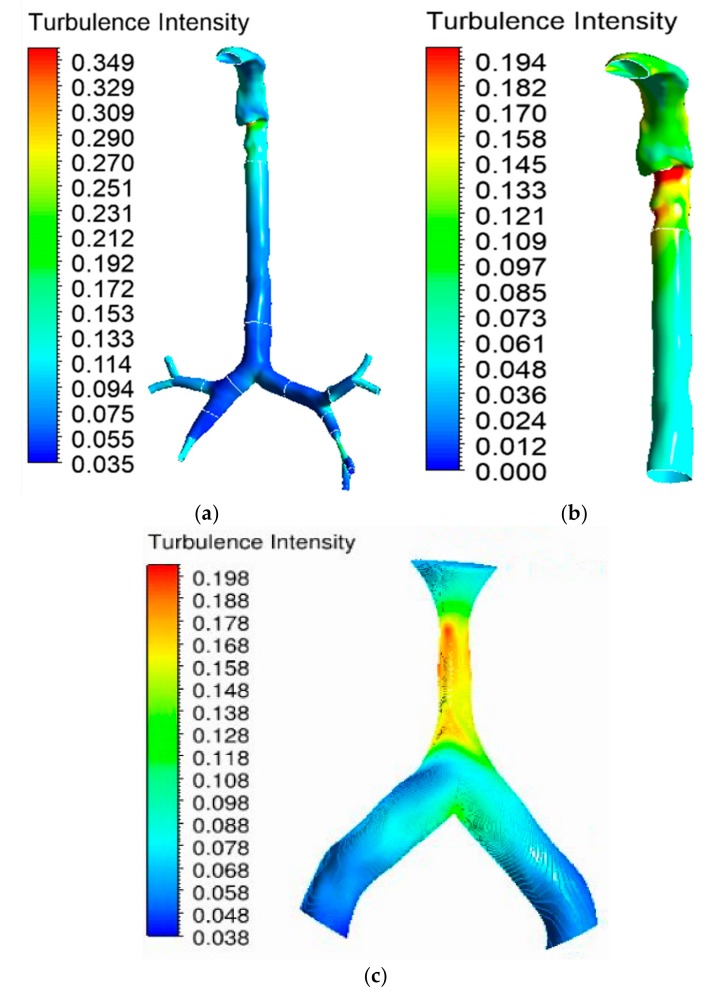
Turbulence intensity at 30 lpm for the right stenosis lung model: (**a**) whole model, (**b**) mouth–throat and trachea (intensity range rescaled), and (**c**) right lung stenosis area.

Turbulence in the oral airway determines the overall airflow pattern throughout the airways. The turbulence intensity contour at the mouth–throat and tracheobronchial airways was calculated for the 30 lpm flow condition. Airflow becomes locally turbulent at a flow rate of ≥30 lpm [38]. Figure 11a shows the turbulence intensity for the healthy lung model. The maximum turbulence intensity was found behind the mouth–throat airway at the upper trachea. The highly asymmetric shape of the mouth–throat passage and strong changes in curvature influence the flow pattern in the mouth–throat area. Figure 11b shows how flow becomes locally turbulent in the mouth–throat and upper tracheal wall area. The intensity range was rescaled for Figure 11b, where the maximum-intensity zone is at the upper tracheal airway. Figure 11c shows the turbulence intensity at the right upper bifurcation of the healthy lung, where maximum intensity was found at the carinal angle (bifurcation) area. For the left and right stenosis models, the overall turbulence intensity is similar to that of the healthy model. The maximum turbulence intensity was found to be identical for all lung models. Figure 12 and Figure 13 show the intensity contours for stenosis conditions at a 30 lpm flow rate. The turbulence intensity at the right stenosis and left stenosis was found to be different from that of the healthy model. Turbulence intensity at the right stenosis is also higher than at the left stenosis. The constricted conduit sections of the right and left stenosis models influence the flow pattern, and the turbulence intensity increases. 

**Figure 14:**
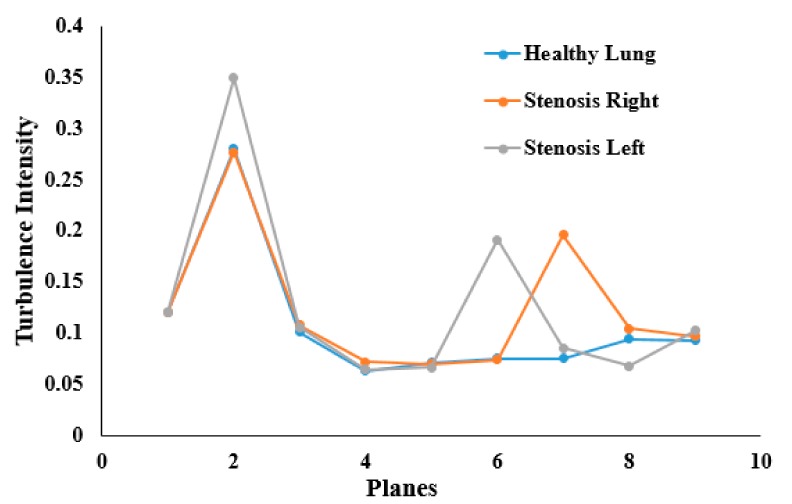
Turbulence intensity at different plane positions of the whole lung models at 30 lpm.

Turbulence intensity values were calculated at different sections of the mouth–throat and tracheobronchial airways. Figure 14 reports the intensity at nine different positions of the healthy and stenosis models. The intensity plot depicts turbulence fluctuation reaching its peak at the mouth–throat area (Plane 2). For the healthy model, turbulence intensity drops beyond the mouth–throat and remains low. On the contrary, the turbulence dispersion at the stenosis section of the right and left stenosis models has a second maximum at the stenosis. The turbulence intensity at the right stenosis was found to be only marginally higher than that at the left stenosis. 

**Figure 15:**
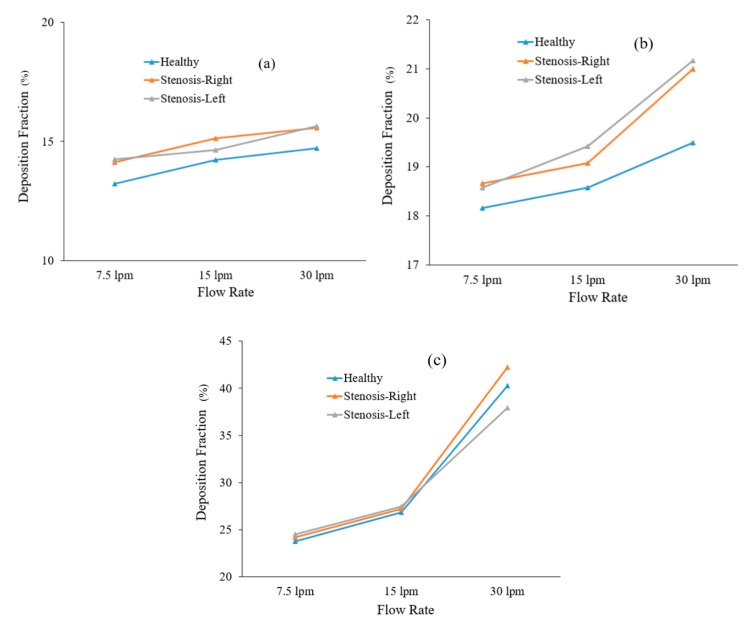
Deposition fraction of different-diameter particles in healthy and stenosis airways: (**a**) 2.5 µm diameter, (**b**) 5 µm diameter, and (**c**) 10 µm diameter.

Particle transport scenarios for various diameter particles were investigated, and the deposition fractions at different flow rates are shown in Figure 15. We found an increasing deposition fraction trend with flow rate and particle diameter and the published literature supports the findings of this study [40]. The total deposition fraction of the various-diameter particles in the healthy lung model was found to be lower than that in the stenosis airway model, irrespective of inhalation conditions. The reduction in airway volume increases the overall deposition fraction. 

**Figure 16:**
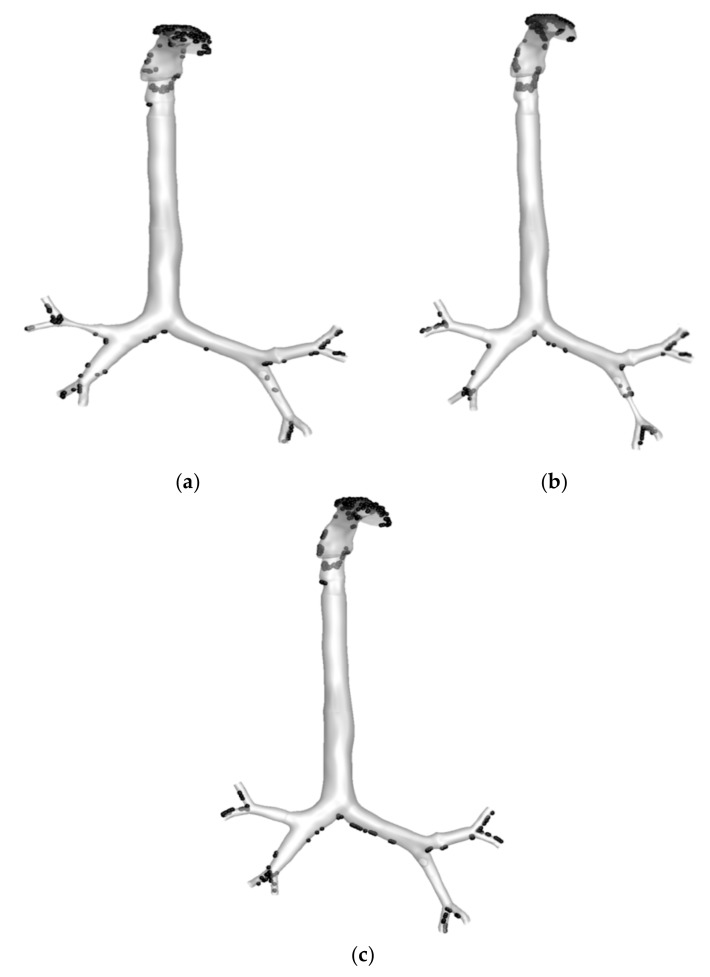
Deposition fraction of 2.5 µm particles in healthy and stenosis airways at 30 lpm: (**a**) right stenosis, (**b**) left stenosis, and (**c**) healthy airways.

The deposition of 2.5 µm particles under the 30 lpm inlet condition was investigated. Figure 16 reports the overall trapped particles in the different stenosis models. Under the 30 lpm condition, particles are commonly trapped at the mouth–throat section of the whole model. The highly irregular and complex shape of the mouth–throat and corresponding flow behavior influence the deposition at the upper section of the model. Figure 16a shows that the deposition in the right upper bifurcation area after the stenosis section is higher than that in the corresponding areas of the healthy and left stenosis models. At the stenosis section, the velocity magnitude increased, and particles hit the bifurcation wall once the particles crossed the stenosis section. The higher velocity influenced the particle trajectory, and the sudden change in the airway curvature increased the deposition at the bifurcation area. Figure 16b shows the deposition pattern for the left stenosis model. The deposition pattern at the stenosis section of Figure 16b is different than that for the two other models. The overall deposition for healthy and stenosis airways shows that the stenosis airway influences the particle transport to the neighboring airways.

## 6. Limitations of the Study

- Outflow outlet conditions were used in this study. In real life, at the third or fourth generation of the airway, there should be some pressure which is unknown.
- No dynamic wall motion was considered for this study.
- Only inhalation was considered for the airflow and particle transport.
- Airway inflammation was not considered.

## 7. Conclusions

Airflow and particle transport simulations were performed in this study. Calculations were made for three different flow rates and different-sized particles. We conclude the following from the numerical simulation study:

- The airflow velocity fields at the mouth–throat and trachea were found to be complex. The airflow velocity in the right and left stenosis models increased significantly over that in the healthy lung model.
- Pressure in the mouth–throat region was maximum, while the stenosis areas showed lower pressure. The highest pressure drop was recorded at the right stenosis, and the highest pressure was observed in the upper trachea (Plane 3) in the left stenosis lung. The overall pressure drop followed a nonlinear trend in all models.
- Wall shear at the mouth–throat and stenosis areas was found to be higher than that in other parts of the lung. Wall shear in the right stenosis model was found to be higher than that in the left stenosis model.
- Turbulence intensity in the whole lung was found to be similar in all three models. However, local turbulence intensity at the stenosis areas was found to be different. The right stenosis model showed a slightly elevated turbulence intensity at the stenosis area when compared to the left stenosis model.
- The deposition fraction for healthy and stenosis airways for various-diameter particles increased with the flow rate. The overall deposition fraction in stenosis airways was higher than that in the healthy airway model.

The presented numerical simulation study was based on principles of particle movement and deposition in a normal human lung and under abnormal pulmonary stenosis. The analysis, carried out on three different models, included pressure drop, velocity profiling, and particle deposition. In this study we analyzed airflow and particle movement for 25% airway stenosis at the selected areas. This study contributes to our understanding of the effects of stenosis on airflow and particle movement in the human lung. The comprehensive pressure analysis increases our understanding of safe airway intervention and could potentially help the clinical process of mechanical ventilation. The particle transport study increases our knowledge of drug aerosol transport through stenosis airways and could help clinical targeted drug delivery to the affected airways. Under different disease conditions like asthma, a respiratory condition characterized by the obstruction of distal bronchi that are primarily related to the airways, the percentage of stenosis could be different. Moreover, the impact of fluids has to be considered in a more comprehensive model. In the foreseeable future, patient-specific lung function will be studied.
